# Effect of Dietary Phytase Supplementation on Bone and Hyaline Cartilage Development of Broilers Fed with Organically Complexed Copper in a Cu-Deficient Diet

**DOI:** 10.1007/s12011-017-1092-1

**Published:** 2017-07-15

**Authors:** Siemowit Muszyński, Ewa Tomaszewska, Małgorzata Kwiecień, Piotr Dobrowolski, Agnieszka Tomczyk

**Affiliations:** 1 Department of Physics, Faculty of Production Engineering, University of Life Sciences in Lublin, Akademicka 13, 20-950 Lublin, Poland; 2 Department of Animal Physiology, Faculty of Veterinary Medicine, University of Life Sciences in Lublin, Akademicka 12, 20-950 Lublin, Poland; 3 Institute of Animal Nutrition and Bromatology, University of Life Sciences in Lublin, Akademicka 13, 20-950 Lublin, Poland; 4 Department of Comparative Anatomy and Anthropology, Maria Curie-Skłodowska University, Akademicka 19, 20-033 Lublin, Poland

**Keywords:** Organic copper, Phytase, Bone histomorphometry, Mechanical testing, Broiler chicken

## Abstract

Tibial mechanical, chemical, and histomorphometrical traits were investigated for growing male Ross 308 broiler chickens fed diets that had copper (Cu) from organic source at a lowered level of 25% of the daily requirement (4 mg kg^−1^ of a premix) with or without phytase. Dietary treatments were control non-copper, non-phytase group (0 Suppl); 4 mg kg^−1^ Cu non-phytase group (25%Cu); and 4 mg kg^−1^ Cu + 500 FTU kg^−1^ phytase group (25%Cu + phyt). The results show that birds fed with the addition of phytase exhibited improved weight gain and final body weight and had increased serum IGF-1 and osteocalcin concentrations. The serum concentration of Cu and P did not differ between groups; however, Ca concentration decreased in the 25%Cu + phyt group when compared to the 25%Cu group. Added Cu increased bone Ca, P, Cu, and ash content in Cu-supplemented groups, but bone weight and length increased only by the addition of phytase. Bone geometry, yield, and ultimate strengths were affected by Cu and phytase addition. A decrease of the elastic stress and ultimate stress of the tibia in Cu-supplemented groups was observed. The histomorphometric analysis showed a positive effect of Cu supplementation on real bone volume and trabecular thickness in the tibia metaphyseal trabeculae; additionally, phytase increased the trabeculea number. The supplementation with Cu significantly increased the total articular cartilage and growth plate cartilage thickness; however, the changes in thickness of particular zones were dependent upon phytase addition. In summary, dietary Cu supplements given to growing broilers with Cu in their diet restricted to 25% of the daily requirement had a positive effect on bone metabolism, and phytase supplementation additionally improved cartilage development.

## Introduction

Copper (Cu) as an essential trace element has many physiological functions in animals and humans, including skeletal mineralization, erythropoiesis, leukopoiesis, connective tissue synthesis, myelin formation, melanin pigment synthesis, catecholamine metabolism, thermal regulation, cholesterol metabolism, immune function, cardiac function, and glucose metabolic regulation [1, 2]. It is known that Cu takes part in bone metabolism as an essential co-factor needed for the action of lysyl oxidase [3]. This Cu-dependent enzyme initiates the critical process of covalent cross-linkage formation in elastin and collagen in bones and other connective tissues [1, 4]. Inhibition of lysyl oxidase enzyme activity results in osteolathyrism and negatively affects bone and cartilage formation [5]. Furthermore, studies on Cu supplementation indicate that Cu deficiency leads to bone loss (osteopenia or osteoporosis), demineralization, a failure of ossification of growth centers, and neuropenia [1, 2]. It is caused mainly by the decreased function of osteoblasts (bone tissue-forming cells) because the action of osteoclasts (bone tissue-resorbing cells) remains unaffected [3]. All of these phenomena lead to the decrease in bone mechanical strength and result in consequent fractures [4, 6].

Trace minerals are essential in the diets of poultry because they participate in the biochemical processes required for the maintenance of normal growth and development, including bone and eggshell formation and development of the avian embryo [7]. Moreover, in poultry, there is substantial interest in using Cu as a health promoter as an alternative to antibiotics that can produce equivalent effects on chicken performance [8]. Copper is often added to poultry diets at prophylactic concentrations for its growth-promoting effects by influencing the microbial populations within the gastrointestinal tract [9].

The Cu requirement in the diets of chicken has been established to be 8 mg kg^−1^ [10], but a greater amount of Cu than is required has often been included in commercial broiler diet. The reason for this is that the differences in the concentrations of Cu and its bioavailability in various feed ingredients are as a result of the variations in processing procedures and in the cultivating conditions used for feed ingredients [10]. Therefore, several supplemental Cu sources such as inorganic sources (copper sulfate or carbonate) and organic sources (chelated form with higher Cu bioavailability) have been added to livestock diet, including broilers [6, 11–13]. Chelates are reported to have significantly higher absorption rates from the intestine due to their unique chemical structure as compared to inorganic salts, and it has been shown that supplementation with 4 mg kg^−1^ of Cu from organic sources may be sufficient for normal broiler growth [11]. Furthermore, organically complexed trace minerals make available alternative pathways for absorption, thus leading to a reduction in the excretion of minerals to the environment [8, 11]. Therefore, it seems that the use of a mineral chelate allows for maintaining performance while concomitantly reducing the mineral content in manure [13, 14].

The skeletal system consists of bones, cartilage, ligaments, and tendons and accounts for about 20% of the body weight of most vertebrates. It has been demonstrated that the use of Cu in the form of glycinate compounds at much reduced amounts as opposed to using sulfate at its recommended dose does not cause deterioration in the physical, mechanical, and morphometric properties of femur in chickens [2, 14, 15]. Thus, with regard to the important role of Cu in the development of bones, it is hypothesized that the use of Cu in the more digestible and assimilable form of chelate might improve the growth and development of the skeletal system in broiler chickens.

Phytic acid is a naturally occurring organic complex found in plants. About 70% of P present in the feedstuffs of plant origin used in the feed for poultry is in phytate form, inaccessible to non-ruminant animals [14]. Phytic acid, as a reactive anion, forms insoluble salts with Cu and other divalent cations [14]. These complexes are indigestible for poultry, and the utilization of microelements bound in phytate form is lowered or impossible [14]. However, it is well known that the use of phytase, an enzyme that hydrolyzes phytate complexes, increases the availability of trace minerals [16, 17]. Studies to quantify the bioavailability of dietary Cu given at marginally deficient amounts from alternative sources of Cu are numerous [2, 6, 11]. However, to date, the relationship between the dietary status of Cu and the presence of phytase on bone health and cartilage development has not been well studied, including on broiler chickens.

With regards to the important role of Cu in bone metabolism during development, the objective of this study was to compare the effects of administrating amino acid Cu complex in growing male Ross 308 chickens fed a Cu-poor diet, which ensured higher Cu bioavailability through enhanced absorption from the intestine, to those fed the same diet but which were additionally enriched with phytase. This was assessed on the basis of the mechanical, geometric, and histomorphometric parameters of the tibia, bone Cu content, and determination of the concentration of hormones of the somatotropic axis in serum. It is hypothesized that the use of Cu in the more assailable form of glycinate chelate might improve the development of the skeletal system in broiler chickens, even if it is administered at the reduced level of 25% of the daily recommended dose. Thus, to evaluate the possible response of broilers to organically complexed Cu and the effect of phytase inclusion into the diet, the present study was conducted with a control diet devoid of both phytase and mineral supplementation of Cu in a trace mineral premix.

## Materials and Methods

The experimental procedures used throughout this study were approved by the Local Ethics Committee on Animal Experimentation of the University of Life Sciences of Lublin, Poland. The birds were maintained in an animal house according to the guidelines of this committee. All efforts were made to minimize the number of animals used as well as their suffering.

### Animals and Experimental Design

A total of 120 1-day-old Ross 308 broiler chickens were obtained from a commercial hatchery. The birds were weighed after hatching and randomly selected to one of three dietary treatments, each group containing 40 chickens. The chickens were assigned to either a control group (the 0 Suppl group; 40 birds divided into 10 pens with 4 birds per pen), or a group fed with lowered level of organic Cu in the form of glycinate chelate (Cu-Gly) as experimental group I (the Cu25% group; 40 birds divided into 10 pens with 4 birds in each pen), or a group fed with lowered level of organic Cu in the form of glycinate chelate simultaneously enriched with phytase as experimental group II (the Cu25% + phyt group; 40 birds divided into 10 pens with 4 birds in each pen). All birds were raised in battery cages (76 × 97 × 45 cm, width × length × height) placed in an environmentally controlled room and kept under standard rearing conditions and air temperature set at the optimal level depending on age. During the first week, the chickens were kept at 33 °C, which was reduced by 2 °C weekly, until the final temperature of 24 °C. The chickens had constant access to fresh water and appropriate feed supplied ad libitum in accordance with this stage of the production cycle (Table 1). To evaluate the growth rate, the birds’ daily body weight gains were recorded. The birds were fed a diet corresponding to the periods of rearing: starter (1–21 days), grower (22–35 days), and finisher (36–42 days). The chickens received a starter diet in the form of crumble, and grower and finisher diets in the form of pellets. At the end of the experiment, 10 birds randomly selected from the control (1 bird from each pen) and experimental groups I and II (1 bird from each pen) were weighed and slaughtered by cutting the carotid arteries. Ten hours before the slaughter, the selected birds were not given feed, but only provided with unlimited access to water.

**Table 1:** Composition and nutritive value of the experimental diet

Ingredients (%)	Starter (1–21 days)	Grower (22–35 days)	Finisher (36–42 days)
Corn	24.5	40.0	40.0
Wheat	42.9	27.9	28.8
Soybean meal^f^	25.0	24.9	22.9
Soybean oil	2.50	3.69	3.98
Phosphate 1—Ca	0.90	0.90	0.81
Fodder chalk	1.40	1.13	1.09
Sodium bicarbonate	0.08	0.08	0.08
NaCl	0.29	0.25	0.26
Premix vitamin (no Cu)	0.50^a^	0.50^b^	0.50^c^
Concentrate protein—fatty^g^	1.00	–	1.00
dl-Methionine 99%	0.30	0.23	0.23
l-Lysine HCl	0.42	0.28	0.27
l-Threonine 99%	0.18	0.13	0.07
The nutritional value of diet			
ME (MJ kg^−1^)^e^	12.7	13.1	13.2
Total protein (%)^d^	21.2	20.4	19.9
Crude fiber (%)^d^	1.64	1.59	1.73
Crude fat (%)^d^	4.57	5.42	5.53
Lysine, total (%)^d^	1.28	1.14	1.08
Met + Cys (%)^d^	0.92	0.81	0.82
Total Ca (%)^d^	0.87	0.79	0.76
Total P (%)^d^	0.65	0.66	0.64
Phytate P (%)^d^	0.32	0.32	0.32
Bioavailable P (%)^e^	0.42	0.41	0.39
Total Ca/P bioavailable^e^	2.12	1.90	1.92
Cu from plants in basal diet (mg kg^−1^)^d^	6.04	6.07	5.91
Fe (mg kg^−1^)^d^	40.31	39.82	38.61
Cu (mg kg^−1^)^d^			
4 mg Cu-Gly	10.28	10.34	10.21
4 mg Cu-Gly + Phyt	11.12	10.87	11.01

^a^Content of vitamins and minerals per 1 kg of starter: Mn 100 mg, I 1 mg, Fe 40 mg, Cu 16 mg, Se 0.15 mg, vitamin A 15000 UI, vitamin D_3_ 5000 UI, vitamin E 75 mg, vitamin K_3_ 4 mg, vitamin B_1_ 3 mg, vitamin B_2_ 8 mg, vitamin B_6_ 5 mg, vitamin B_12_ 0.016 mg, biotin 0.2 mg, folic acid 2 mg, nicotinic acid 60 mg, pantothenic acid 18 mg, choline 1800 mg

^b^Content of vitamins and minerals per 1 kg of grower: Mn 100 mg, I 1 mg, Fe 40 mg, Cu 16 mg, Se 0.15 mg, vitamin A 12000 UI, vitamin D_3_ 5000 UI, vitamin E 50 mg, vitamin K_3_ 3 mg, vitamin B_1_ 2 mg, vitamin B_2_ 6 mg, vitamin B_6_ 4 mg, vitamin B_12_ 0.016 μg, biotin 0.2 mg, folic acid 1.75 mg, nicotinic acid 60 mg, pantothenic acid 18 mg, choline 1600 mg

^c^Content of vitamins and minerals per 1 kg of finisher: Mn 100 mg, I 1 mg, Fe 40 mg, Cu 16 mg, Se 0.15 mg, vitamin A 12000 UI, vitamin D_3_ 5000 UI, vitamin E 50 mg, vitamin K_3_ 2 mg, vitamin B_1_ 2 mg, vitamin B_2_ 5 mg, vitamin B_6_ 3 mg, vitamin B_12_ 0.011 μg, biotin 0.05 mg, folic acid 1.5 mg, nicotinic acid 35 mg, pantothenic acid 18 mg, choline 1600 mg

^d^Analyzed values

^e^Calculated values

^f^Forty-six percent total protein in dry matter

^g^One kilogram concentrate protein-fatty contains 2% crude fat, 39% crude protein, 10.8 MJ ME

Immediately after slaughter, the tibiae were dissected and cleaned from the remnants of adherent tissues and their weight and length measured. Directly after the measurements, each bone was wrapped in gauze soaked in isotonic saline and frozen at −25 °C for further analyses.

### Supplementation of Cu Amino Acid Chelate and Phytase

The control group (the 0 Suppl group) was fed basal diet supplemented with the premix which did not provide external Cu (0 mg kg^−1^). The experimental diets were formulated by supplementing a corn-wheat-soyabean meal mixture (Table 1) with lowered levels (25% of the total daily recommended amount for Ross 308 broiler, 4 mg kg^−1^) of Cu from Gly-Cu, with or without phytase (500 FTU kg^−1^). The experiment involved the use of Glystar Forte chelate (Arkop Sp. z o.o., Bukowno, Poland) containing 16% of Cu and Ronozyme® HiPhos 6-phytase (DSM Nutritional Products, Mszczonów, Poland) produced by a genetically modified strain of *Aspergillus oryzae*. Application of glycine chelate was in accordance with the EU Directive 1334/2003 [18].

The basal corn-wheat-soybean meal diet (Table 1) containing (by analysis) 6.1 mg kg^−1^ (starter), 6.21 mg kg ^−1^ (grower), and 5.91 mg kg^−1^ (finisher) of Cu from plants as the feed basis was formulated to meet or exceed nutritional requirements [10]. The amount of Cu in the premix was based on nutritional recommendations for Ross 308 broilers [10, 19], i.e., 16 mg kg^−1^ of Cu, irrespective of its content in the components of the basal diet. According to these recommendations, the Cu content should be the same in all periods of rearing, which was taken into account in the study [10, 19].

The nutrient composition of the basal diet was analyzed using standard methods: total phosphorus, calorimetrically with a Helios Alpha UV-VIS apparatus (Spectronic Unicam, Leeds, UK) [20], phytic phosphorus, by the Frühbeck et al. method [21], and the Cu, Fe, and Ca content in feed samples determined after ashing at 550 °C using the AAS flame technique in a Unicam 939 AA Spectrometer (Shimadzu Corp., Tokyo, Japan) apparatus, according to the methods of AOAC [20].

The amino acid composition in the diet was determined by ion exchange chromatography using an INGOS AAA 400 amino acid analyzer with post-column derivatization of ninhydrin and spectrophotometric detection [20]. Cysteine and methionine (sulfur amino acids) were determined in a separate analysis as described previously [15], according to the method of AOAC [20]. Assimilable lysine was determined based on the difference between total lysine and the so-called residual lysine which did not react with DNFB (dinitrofluorobenzene) [15]. Following this reaction, the tested samples were again subjected to acid hydrolysis [22].

### Serum Biochemical Analyses

Each chicken was fasted for 12 h before blood collection. The blood was collected using standard venipuncture from the brachial vein; next, after clotting at room temperature, it was centrifuged and frozen at −80 °C for further analysis. The blood serum concentrations of copper, calcium, and phosphorus were determined by a colorimetric method using a Metrolab 2300 GL unit (Metrolab SA, Argentina) and sets of biochemical reagents produced by BioMaxima (Lublin, Poland) [20].

### Growth Hormone and Bone Turnover Markers

The serum concentration of chicken growth hormone, insulin-like growth factor 1 (IGF-1), osteocalcin, and leptin were determined using an enzyme-linked immunosorbent assay kit (ELISA; Uscn Life Science Inc. Wuhan, China) with minimum detectable concentrations of 0.056 ng ml^−1^, 7.4 pg ml^−1^, 0.67 pg ml^−1^, and 14.8 pg ml^−1^, respectively.

### Mechanical Properties

The mechanical properties of the tibia were determined for all the groups after 3-h thawing at room temperature using the three-point bending test of bone mid-diaphysis. The mechanical properties were examined on a Zwick Z010 universal testing machine (Zwick GmbH & Co. KG, Ulm, Germany), equipped with a measuring head of operation range up to 10 kN, linked to a computer with testXpert II 3.1 software (Zwick GmbH & Co. KG, Ulm, Germany), registering the relationship between force perpendicular to the longitudinal axis of the bone and the resulting displacement. The distance between the supports was set at 40% of the total bone length. The measuring head loaded bone samples with a constant speed of 10 mm min^−1^ until fracture [23]. The ultimate load was determined as the force causing bone fracture and the yield load as maximal force under an elastic (reversible) deformation of the bone [24]. Moreover, on the basis of measured geometric and mechanical traits, the material properties of the mid-diaphyseal fragment of the bone were calculated. These traits describe the specific mechanical properties of the midshaft cortical tissue and are independent of the bone size and the conditions under which the strength tests were conducted. The bending moment can be described as a yield load adjusted to the bone length, and it indicates the bone elastic load capability [25]. The elastic stress reflects the elastic strength of midshaft cortical bone; the ultimate stress is equal to the maximum stress a bone can withstand in bending before fracture [25].

### Geometric Parameters

The geometric properties such as the cross section area (A), the mean relative wall thickness (MRWT), and the cortical index (CI; defined as the ratio of the thickness of the cortical part to the thickness of the midshaft measured at the middle part of the bone) were estimated on the basis of the horizontal and vertical diameter measurements of the mid-diaphyseal cross section of the bone with the previously described method [26]. Moreover, as during the strength analysis the bone was loaded in the A-P plane, the second (cross-sectional) moment of inertia Ix and the radius of gyration Rg about the medial-lateral (M-L) axis were calculated [25]. The second moment of inertia Ix is not a direct bone geometric trait, but is a critical property in terms of the bone bending rigidity evaluation.

The samples of the proximal end of each bone were subjected to histology as described previously [2]. The site and size (approximately 3 mm in length of both analyzed cartilages) of the areas of interest which were measured were chosen on the basis of motoric properties of the body—the knee joint in particular—as was described previously [27]. Two methods of staining were used: the Goldner’s trichrome to assess the morphology of the growth plate and articular cartilage, and the safranin-O staining to visualize the cartilage proteoglycans [28]. Briefly, the sagittal sections through the middle of the lateral condyle of each tibia were cut strictly according to the previously described method and equipment [27]. Safranin-O staining was applied to the visual assessment of Mankin’s histological and histochemical grading system for evaluation of the articular cartilage [29, 30].

The thickness of the following zones: reserve (I), proliferation (II), hypertrophy (III), and ossification (IV) were measured at four sites along the growth plate cartilage, and an average was calculated as described previously [30]. Similarly, the thickness of the main zones of the articular cartilage, i.e., horizontal (superficial surface, I), transitional (II), and radial (III), was measured as described previously [2].

The Picrosirius red staining (PSR) was employed to assess the morphology of the articular cartilage and to evaluate the distribution of thick (mature) and thin (immature) collagen fibers in the articular cartilage [31–33]. The sections stained with PSR were analyzed using a Leica DM 2500 microscope (Leica Microsystems, Wetzlar, Germany) equipped with filters to provide circularly polarized illumination. Images were documented by a high-resolution digital camera (Leica Microsystems, Wetzlar, Germany).

The bone volume (BV), tissue volume (TV), relative bone volume (BV/TV), trabecular thickness (Tb.Th), trabecular separation (Tb.Sp), trabecular number (Tb.N), and fractal dimension (Fd) of the trabecular bone were measured as described previously [28].

### Ca, P, and Ash Content in Bone

After evaluating the strength and structural properties, the bones were defatted, dried at 105 °C to a constant mass, and finally mineralized in a muffle furnace at 500 °C [2, 22]. The content of the mineral components (Ca, P, Cu) of the bones was determined by an atomic absorption spectrometry using a Unicam 939/959 apparatus [20]. The percentage of bone ash and the content of Ca, P, and Cu in the bone were calculated as part of components from the crude ash.

### Statistical Analysis

All results are expressed as mean ± SD (standard deviation). The differences between the means were tested with one-way ANOVA and post hoc Tukey’s HSD test as the correction for multiple comparisons. Normal distribution of data was examined using the Shapiro-Wilk W-test, and equality of variance was tested by the Brown-Forsythe test. A *P* value of less than 0.05 was considered statistically significant. All statistical analyses were carried out by means of Statistica 12 software (StatSoft, Inc., Tulsa, OK, USA; http://www.statsoft.com).

## Results

### Body Weight

The initial body weights of the control and birds treated with the organic Cu form (regardless of the presence of phytase) were similar (Table 2). At the end of the experiment, chickens fed Cu-poor diet supplemented with the phytase (the Cu25% + phyt group) weighed significantly more than the birds from the control group (the 0 Suppl group) and the Cu25% group (Table 2). Daily weight gain was also significantly higher in the Cu25% + phyt group compared to the control and the Cu25% groups (Table 2).

**Table 2:** The body weight (initial and 42-day-old as final body weight) of control broilers and supplemented with Cu in 25% of daily demand dependently on phytase supplementation

Group	Number	Body weight (g)		Daily weight gain (g)
		Initial body weight	Final body weight	
0 Suppl	10	44.1 ± 1.0	2262 ± 127a	49.4 ± 4.3a
Cu25%	10	43.8 ± 1.0	2281 ± 144a	53.3 ± 3.4a
Cu25% + phyt	10	44.0 ± 0.8	2716 ± 160b	63.6 ± 3.8b
SEM		0.17	46	1.31
*P* value		*P* = 0.775	*P* < 0.001	*P* < 0.001

Mean values in columns with different lowercase letters differ significantly at *P* < 0.05; data given are mean ± SD (standard deviation)

*SEM* standard error of the mean, *0 Suppl* the control group without received Cu in premix, *Cu25%* the group received Cu in 25% of daily demand from Cu-Gly, *Cu25% + phyt* the group received Cu in 25% of daily demand from Cu-Gly with phytase

### The Content of Ca, Total P, and Cu in Blood Serum

**Fig. 1:**
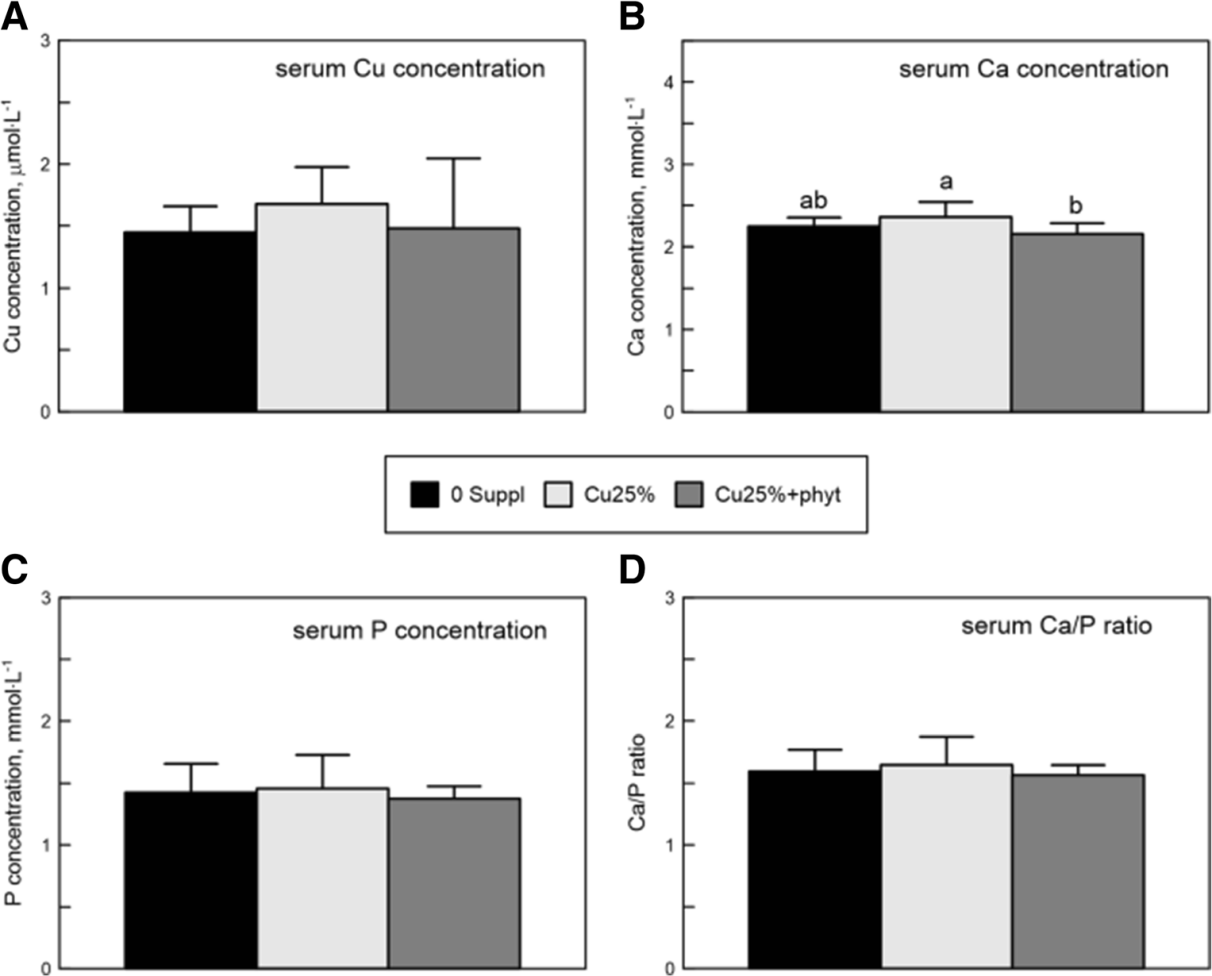
The serum concentration of calcium (Ca), copper (Cu), phosphorus (P), and Ca/P ratio in 42-day-old chickens treated with Cu in organic (Cu-Gly) form dependently on phytase supplementation. Data given are mean ± SD. **a**, **b** *Values with different letters* differ significantly at *P* < 0.05. *0 Suppl*—the control group without received Cu in premix. *Cu25%*—the group received Cu in 25% of daily demand from Cu-Gly. *Cu25% + phyt*—the group received Cu in 25% of daily demand from Cu-Gly with phytase

The Cu serum concentration of the birds from both experimental groups (irrespective of the phytase supplementation) reached similar values as those in the control group (Fig. 1). Similarly, total P serum concentration was similar in the control chickens and birds supplemented with the Cu-Gly, regardless of the phytase addition (Fig. 1). The Ca serum concentration of the chickens from the Cu25% + phyt group was lower compared to the control values noted in chickens from the Cu25% group (Fig. 1). However, the serum Ca/P ratio did not differ between groups (Fig. 1).

### The Content of P, Ca, and Cu in Bone

**Fig. 2:**
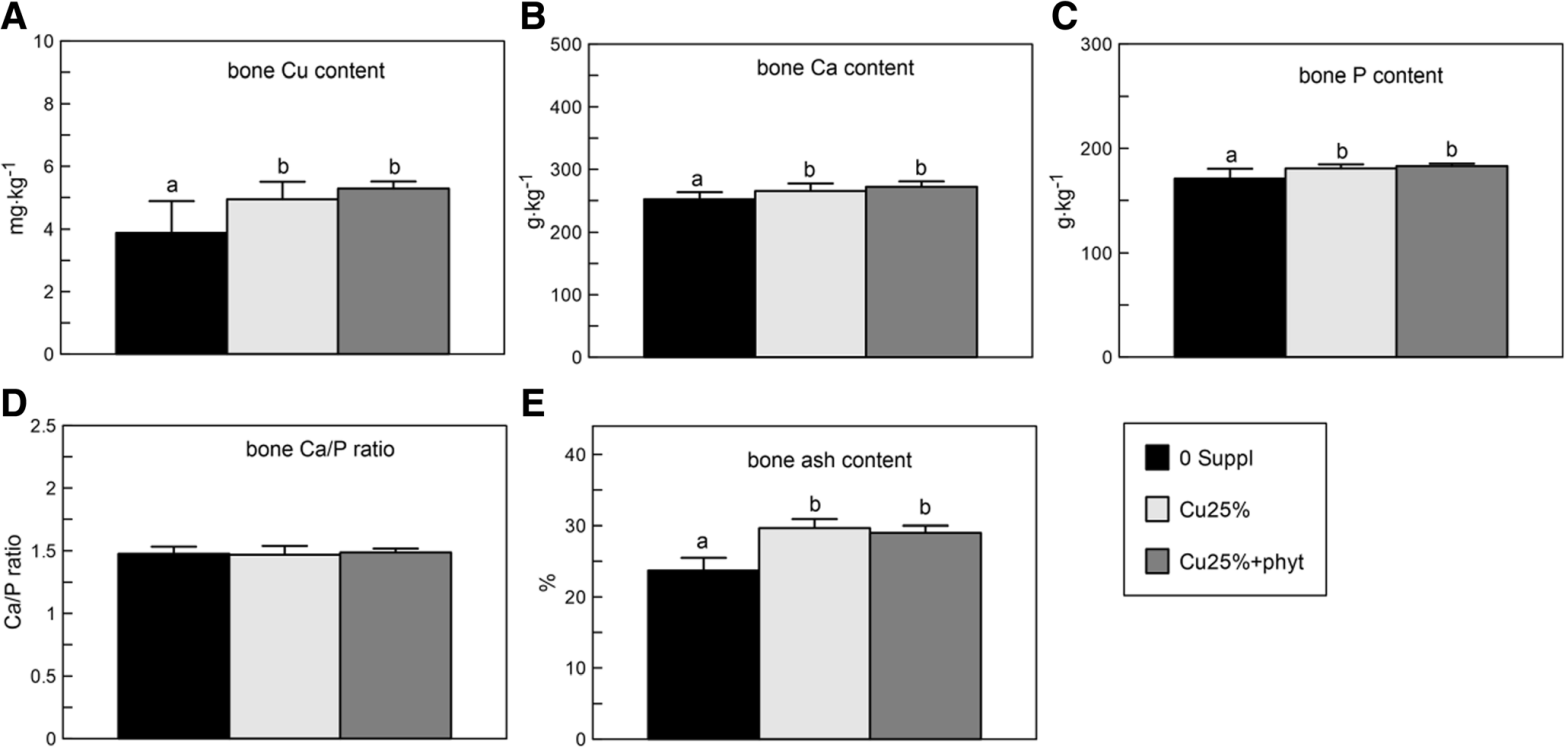
The bone content of calcium (Ca), phosphorus (P), copper (Cu), crude ash, and bone Ca/P ratio in 42-day-old chickens treated with Cu in organic (Cu-Gly) form dependently on phytase supplementation. Data given are mean ± SD. **a**, **b** *Values with different letters* differ significantly at *P* < 0.05. The description of the groups as in Fig. 1

The bone P, Ca, and bone crude ash content in the chickens supplemented with Cu in the Cu-Gly form in the Cu-poor diet, irrespective of the phytase presence, was higher compared to the control values reached in the 0 Suppl group (Fig. 2). However, the bone Ca/P ratio did not differ between groups (Fig. 2). The bone Cu content in both groups supplemented with Cu in 25% of daily demand was higher compared to the control chickens which were fed the Cu-deprived diet (Fig. 2).

### Bone Morphology, Geometry, and Mechanical Properties

The intake of Cu in the Cu-Gly form at the concentration of 25% of daily requirement with the addition of phytase increased the bone weight, length, and weight/length ratio compared to the other groups (Table 3). Moreover, Cu-Gly administration at 25% of daily requirement significantly altered all measured diameters; however, the greatest increases were noted in the group additionally supplemented with phytase (Table 3). As a result, the chickens fed the Cu-poor diet irrespective of the phytase addition had an increased cross section area as compared to the control chickens. However, the mean relative wall thickness decreased in the Cu25% group, while the cortical index increased in the Cu25% + phyt group compared to the control 0 Suppl group (Table 3). The change of the cross section dimensions of the midshaft resulted in a significant change of the values of the midshaft volume and the cross-sectional moment of inertia in both groups fed the Cu-poor diet (with the highest values in the group with phytase addition) when compared to control birds fed the Cu-deprived diet (Table 3). Similarly, an increase in the radius of gyration of both experimental groups was observed.

**Table 3:** The physical, mechanical, and geometric properties of tibia obtained from 42-day-old broilers in the control group and supplemented with Cu in 25% of daily demand dependently on phytase supplementation

Item	Group			SEM	*P* value
	0 Suppl (*n* = 10)	Cu25% (*n* = 10)	Cu25% + phyt (*n* = 10)		
Bone general properties					
Bone weight (g)	19.4a	20.1a	26.2b	0.9	*P* < 0.001
Bone length (mm)	110a	110a	115b	1	*P* < 0.001
Bone weight/bone length (g mm^−1^)	0.176a	0.182a	0.228b	0.01	*P* < 0.001
Bone geometrical properties					
Horizontal internal diameter h (mm)	4.41a	5.08b	5.76c	0.20	*P* < 0.001
Horizontal external diameter H (mm)	6.83a	8.62b	11.18c	0.50	*P* < 0.001
Vertical internal diameter b (mm)	2.07a	4.46b	4.81b	0.33	*P* < 0.001
Vertical external diameter B (mm)	4.52a	7.44b	8.70c	0.49	*P* < 0.001
Cross section area A (mm^2^)	17.1a	32.6b	55.0c	4.5	*P* < 0.001
Mean relative wall thickness MRWT (–)	0.86a	0.71b	0.89a	0.04	*P* = 0.009
Cortical index CI (%)	44.3ab	40.9a	46.3b	1.1	*P* = 0.024
Midshaft volume (cm^3^)	0.76a	1.44b	2.53c	0.21	*P* < 0.001
Moment of inertia Ix (mm^4^)	30a	157b	348c	40	*P* < 0.001
Index of gyration Rg (mm)	1.31a	2.16b	2.45c	0.14	*P* < 0.001
Bone mechanical properties					
Yield strength (N)	149a	158a	383b	29	*P* < 0.001
Ultimate strength (N)	239a	248a	549b	40	*P* < 0.001
Elastic stress (MPa)	133a	46b	61b	11	*P* < 0.001
Ultimate stress (MPa)	215a	69b	86b	18	*P* < 0.001
Bending moment (N·m)	1.64a	1.75a	4.41b	0.35	*P* < 0.001

Mean values in rows with different lowercase letters differ significantly at *P* < 0.05; data given are means

*SEM* standard error of the mean, *0 Suppl* the control group without received Cu in premix, *Cu25%* the group received Cu in 25% of daily demand from Cu-Gly, *Cu25% + phyt* the group received Cu in 25% of daily demand from Cu-Gly with phytase

The addition of phytase in the Cu-poor diet resulted in an increase in the yield strength, the ultimate strength, and the bending moment compared to the control diet (Cu-deprived) and the Cu-poor diet without the phytase. However, for both experimental groups, the Cu supplementation led to a significant decrease of the elastic stress and the ultimate stress of the midshaft cortical bone (Table 3).

### Bone Histomorphometry

The microscopic assessment of cancellous bone in both experimental groups supplemented with Cu-Gly at the concentration of 25% of the daily requirement showed a significant increase in the real bone volume (BV/TV), the mean (Tb.Th mean), and the maximal (Tb.Th max) trabecular thickness (Table 4). However, an increase in the mean (Tb.Sp mean) and the maximal (Tb.Sp max) trabecular space in the Cu25% group when compared to the control group was noted while the phytase addition resulted in a decrease of the Tb.Sp mean and the Tb.Sp max (Table 4). Moreover, in the Cu25% + phyt group, an increase in the trabecula number (Tb.N) was observed. The fractal dimension (Fd) of the trabecular bone increased significantly in the Cu25% + phyt group and decreased in the Cu25% group when compared to the control group.

**Table 4:** The histomorphometrical parameters of trabeculae of cancellous bone in tibia obtained from 42-day-old broilers in the control group and supplemented with Cu in 25% of daily demand dependently on phytase supplementation

Item	Group			SEM	*P* value
	0 Suppl (*n* = 10)	Cu25% (*n* = 10)	Cu25% + phyt (*n* = 10)		
BV/TV (%)	16.2a	18.2b	21.5b	0.08	*P* < 0.001
Tb.Th mean (μm)	33.8a	55.5b	53.4b	1.69	*P* = 0.023
Tb.Th max (μm)	117a	150b	148b	7.45	*P* < 0.001
Tb.Sp mean (μm)	186a	287b	132a	13.00	*P* < 0.001
Tb.Sp max (μm)	597ab	696b	381a	39.70	*P* = 0.012
Fd (–)	1.54b	1.46a	1.62c	0.016	*P* < 0.001
Tb.N (mm^−1^)	4.75a	4.07a	6.13b	0.62	*P* < 0.001

Mean values in rows with different lowercase letters differ significantly at *P* < 0.05; data given are means

*SEM* standard error of the mean, *BV/TV* relative bone volume, *Tb.Th* trabecular thickness, *Tb.Sp* trabecular separation, *Fd* fractal dimension of trabecular bone, *Tb.N* trabecular number, *0 Suppl* the control group without received Cu in premix, *Cu25%* the group received Cu in 25% of daily demand from Cu-Gly, *Cu25% + phyt* the group received Cu in 25% of daily demand from Cu-Gly with phytase

### Morphology of the Articular and Growth Plate Cartilages

**Fig. 3:**
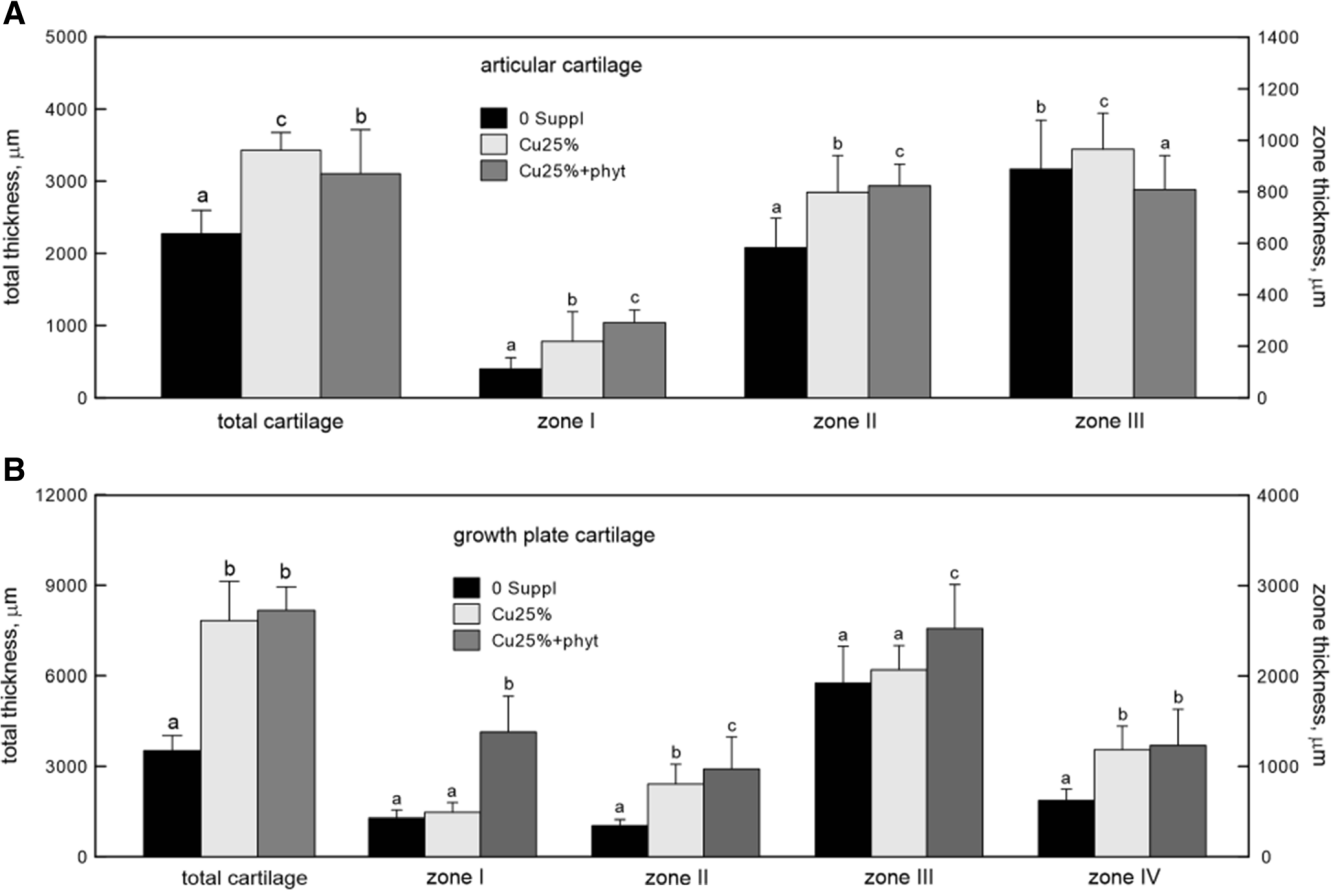
The morphology of the articular cartilage and the growth plate of tibia obtained from 42-day-old chickens treated with Cu in organic (Cu-Gly) form dependently on phytase supplementation. Data given are mean ± SD. **a**, **b** *Values with different letters* differ significantly at *P* < 0.05. The description of the groups as in Fig. 1

The examined joints had no visible lesions or degenerative changes. The supplementation of Cu-Gly to the feed of growing chickens (regardless of phytase addition) significantly increased the total, I, and II zones of the articular cartilage, while the greatest change was observed in the group fed with the phytase additive (Fig. 3). Zone III became wider in the Cu25% group and narrowed in the Cu25% + phyt group when compared to the control group. As a result, the articular cartilage total thickness increased in both experimental groups with the most significant increase being observed in the Cu25% group supplemented with Cu without the addition of phytase (Fig. 3).

Furthermore, the addition of phytase to the Cu-poor diet in the Cu25% + phyt group resulted in an increase of the total and all particular zones of the growth plate cartilage as compared to the control group and in zone I and zone III when compared to the birds fed the Cu-poor diet without the phytase additive (Fig. 3). The Cu-poor diet without the phytase addition resulted in an increase of the total, zone II, and zone IV thickness when compared to the control 0 Suppl group (Fig. 3).

### Proteoglycan Content in the Articular Cartilage

**Fig. 4:**
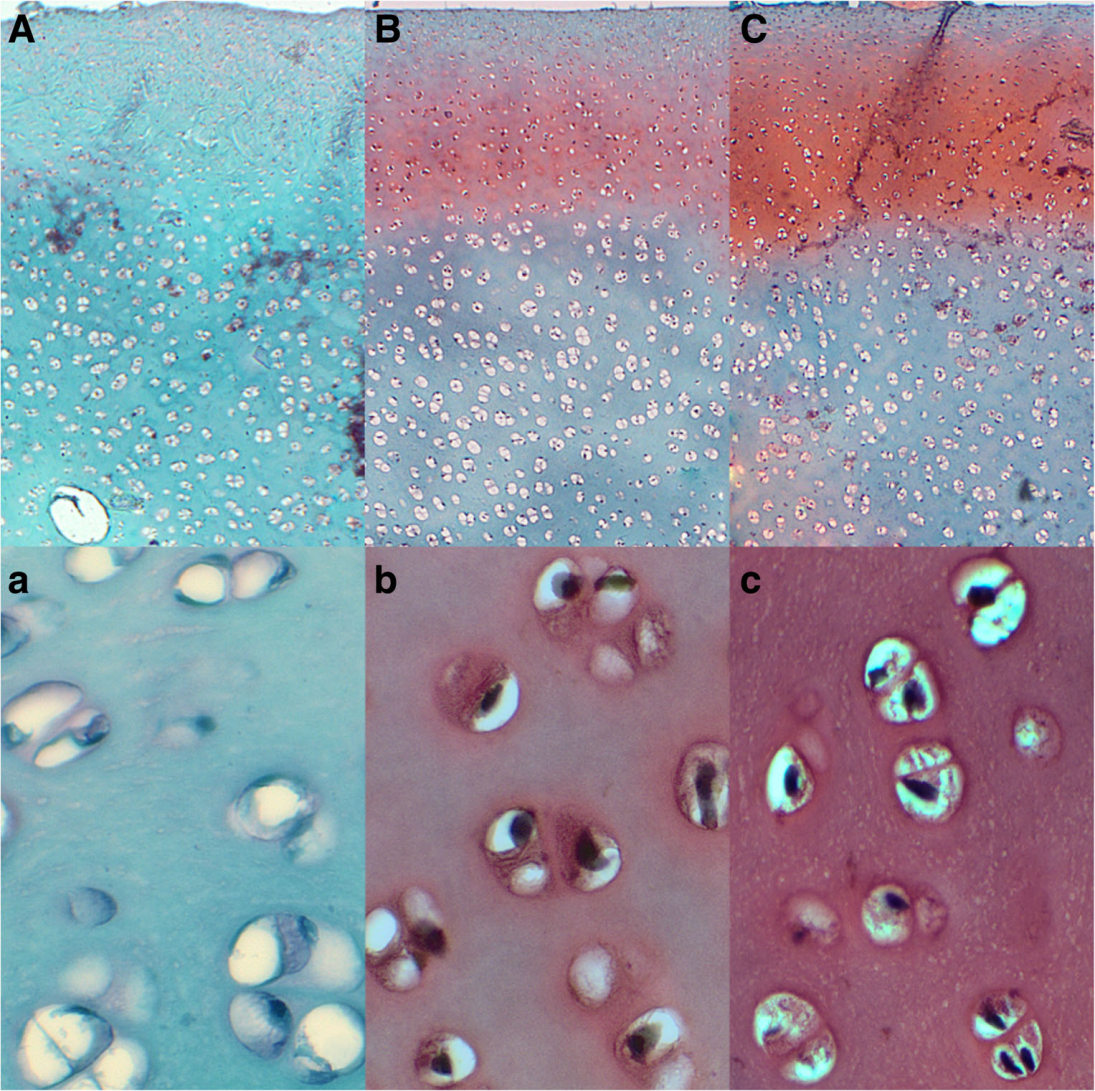
The representative images of safranin-O staining carried out on formaldehyde-fixed sections from the tibial articular cartilage of 42-day-old chickens treated with Cu in organic (Cu-Gly) form dependently on phytase supplementation. The cartilage from the control group displayed a very low proteoglycan content (displaying weaker staining pattern), while the chickens treated with the Cu-Gly at the concentration of 25% of daily demand demonstrated higher but not strong staining patterns linked with a higher content of proteoglycans. The most intensive staining with safranin-O was observed around chondrocytes. Vertical section of zone I and upper part of zone II of the tibial articular cartilage from the control group fed the Cu-deficient control diet (**a**), the Cu25% + phyt group (**b**), and the Cu25% + phyt group (**c**). Magnification ×200. The description of the groups as in Fig. 1

In all examined groups, the osteochondral junction was intact and the surface of the cartilage was smooth without irregularities according to Mankin’s semiquantitative scoring system (Fig. 4). Proteoglycan staining with SO showed a lower proteoglycan content (displaying a weaker staining pattern) in the cartilage from the control group (Fig. 4a), while the chickens treated with the Cu-Gly at the concentration of 25% of daily requirement demonstrated a higher, but only moderate, staining pattern linked with a higher content of proteoglycans. The concentration of proteoglycans in the Cu-Gly-supplemented groups, irrespective of the phytase addition, exhibited a gradual increase with the distance from the periphery of the cartilage to the end of zone I and loss of safranin-O staining from the beginning of zone II (Fig. 4b, c). The most intensive staining pattern with safranin-O was observed around chondrocytes (Fig. 4b, c). In addition, there was no evident gradient in the safranin-O staining within the control chickens fed the Cu-deprived diet and their articular cartilages had a very poor red staining pattern when compared to the other Cu-Gly-supplemented groups (Fig. 4).

### Distribution of Thick and Thin Collagen Fibers and Proteoglycans in the Articular Cartilage

**Fig. 5:**
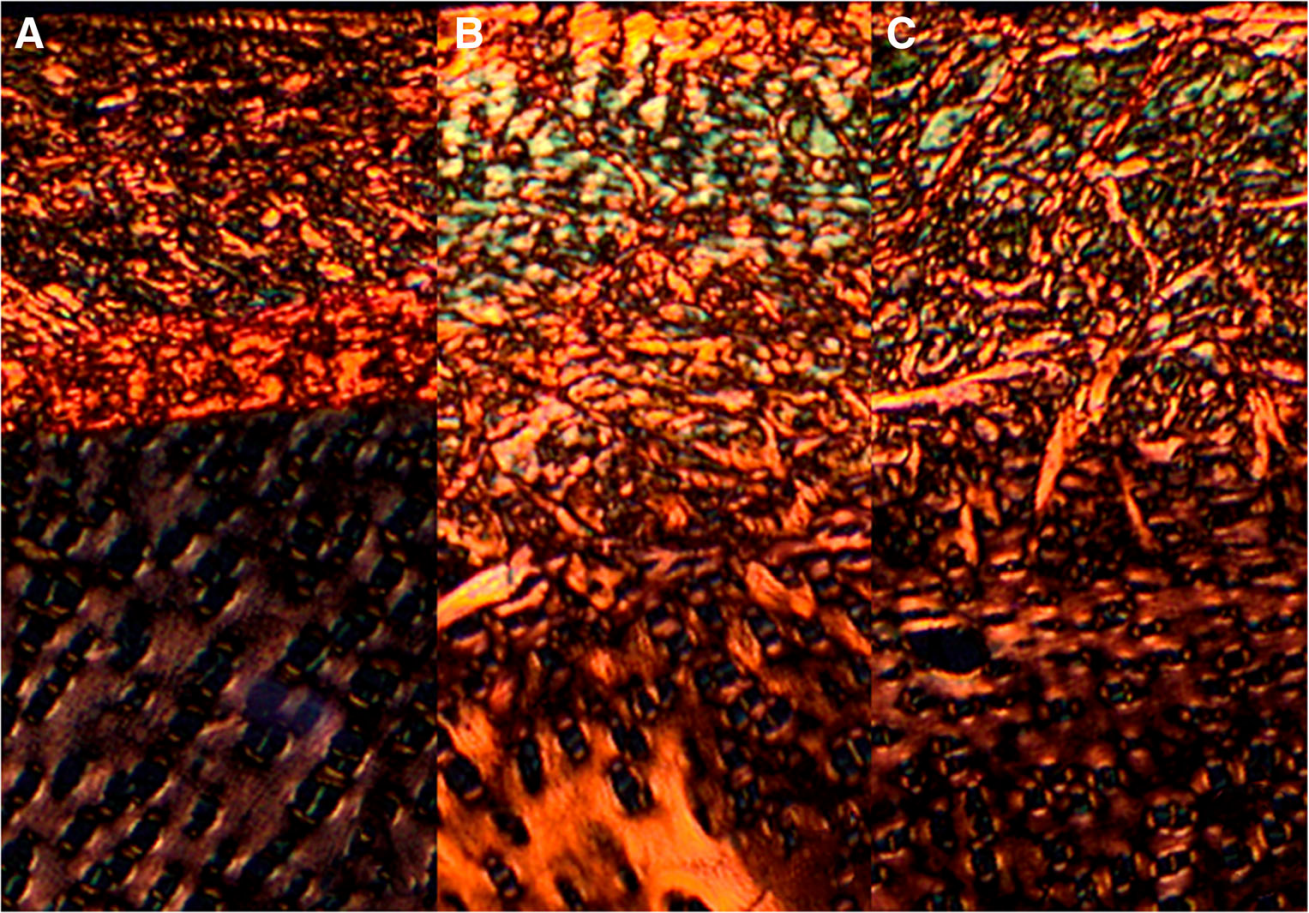
The representative images of PSR staining carried out on formaldehyde-fixed sections from the tibial articular cartilage of 42-day-old chickens treated with Cu in organic (Cu-Gly) form dependently on phytase supplementation. The large collagen fibers are *orange* or *red*, and the thick ones, including reticular fibers, are *green*. Vertical section of zone I of the tibial articular cartilage from the control group fed the Cu-deficient control diet (**a**), the Cu25% + phyt group (**b**), and the Cu25% + phyt group (**c**). Magnification ×200. The description of the groups as in Fig. 1

The structural information obtained from the analysis of fibrous components in the PSR-stained section revealed a difference between large (mature red-orange) and thin (immature green) collagen fibers. The supplementation of Cu (irrespective of phytase addition) enhanced thin (green) and decreased thick (red) fibers in the articular cartilage resulting in greener radial fibers (Fig. 5). Moreover, thin collagen fibers (green) were distinctly discernible in the layer located near the calcified cartilage at the cartilage-bone interface (Fig. 5b, c). An opposite proportion was observed in the control birds.

### Hormonal Analysis

**Fig. 6:**
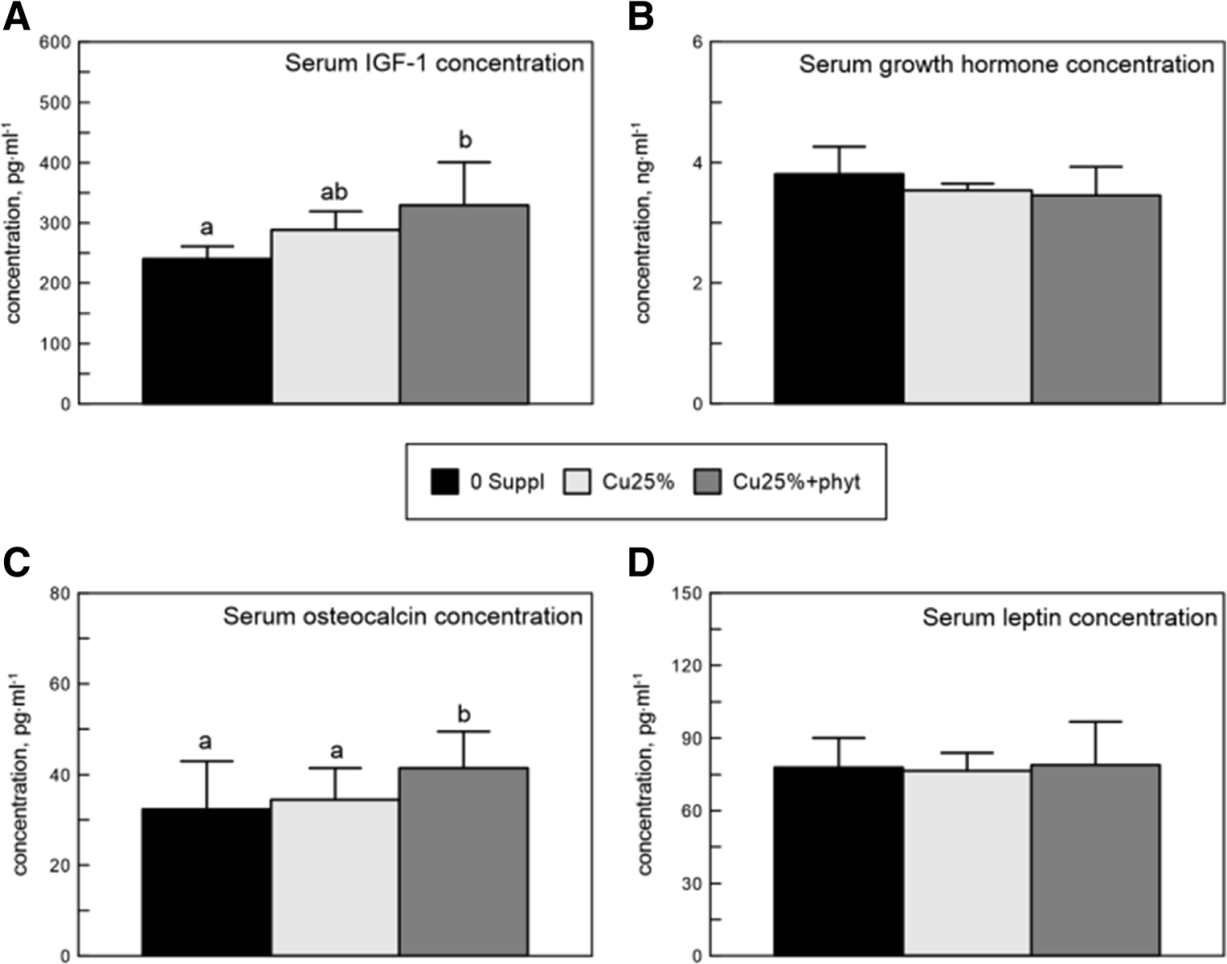
The serum concentration of insulin-like growth factor 1 (IGF-1), growth hormone, osteocalcin, and leptin in 42-day-old chickens treated with Cu in organic (Cu-Gly) form dependently on phytase supplementation. Data given are mean ± SD. **a**, **b** *Values with different letters* differ significantly at *P* < 0.05. The description of the groups as in Fig. 1

The results of the analysis of the growth hormone, leptin, and bone turnover markers are presented in Fig. 6. It was shown that the addition of phytase to the Cu-poor diet for Ross 308 broiler increased the concentration of osteocalcin compared to the other groups, while the concentration of IGF-1 increased only when compared to the control Suppl 0 group (Fig. 6). The experimental diets did not influence the serum concentration of other examined hormones, namely, growth hormone and leptin.

## Discussion

Adequate Cu intake in poultry production is necessary to not only reach genetically optimal growth but also to maintain proper function of the skeletal system, which is an important mineral source and which provides structural support for well-muscled and fast-growing breeds [14, 25]. For this reason, the overloading of long bones could result in locomotion problems that have a negative impact on the performance and welfare of the intensively reared animals and could adversely affect the profitability of livestock production [34]. Additionally, to prevent other health problems associated with rapid growth, the broilers of the fast-growing breeds are fed restricted amounts of feed, especially during the rearing period [35, 36], which can lead to aggressive behavior around feeders [37]. The mechanisms for long bone growth are similar across most animal species, but the growth rate is the fastest in the proximal tibia of domestic fowl [38]. This, in combination with the general weakness of the leg and other leg abnormalities in intensively reared poultry, can deprive the birds of locomotive freedom, leading to suffering, discomfort, fear, and distress during rearing [34]. The syndromes that cause lameness in the birds of a flock can affect one or several musculoskeletal tissues of a single bird or of the whole flock at the same time [39]. Weak bones or tendons result in, e.g., dyschondroplasia, osteochondrosis, or bone loss. Thus, the potential beneficial effects of supplementation with trace elements in organic form on bone health are being increasingly investigated [15, 28, 40]. Such studies are necessary because supplementation with chelated forms of trace elements is an attractive strategy deserving further evaluation in poultry breeding as traditional inorganic mineral salts are often used at levels higher than the recommended dosage in order to avoid trace mineral deficiency [28]. Nevertheless, studies concerning how the skeletal system will adapt to a Cu-poor diet are still limited and further studies are therefore required, especially given that phytase is studied mainly in relation to the bioavailability of P [41].

Our study showed that phytase addition to broilers supplemented with 4 mg kg^−1^ of organic Cu (the Cu25% + phyt group) increased the final body weight, growth rate, and percentage tibia ash as compared to the broilers whose diets were devoid of copper (the control 0 Suppl group) or those without added phytase (the Cu25% group). It was not supported by other studies performed on broiler chickens that were fed control Cu-deficient diets, where increase of body weight after Cu supplementation was observed at lowered levels [4, 42]. Furthermore, Cu bone content increased in both Cu-supplemented groups when compared to the control 0 Suppl group. These findings differed from those presented by Bao et al. [42], where supplementation with organic Cu at 50% of the recommended dose had no effect on the total Cu content of tibia when compared to the Cu-deficient control group. Our results also did not support studies showing that dietary phytase supplementation improved mineral availability (P, Ca, Cu, Mg, Fe) or Ca retention [43]. Our experimental diets containing Cu at 25% of the recommended dose increased Cu, Ca, P, and ash content in bone irrespective of phytase addition, while the serum Ca concentration was decreased by phytase supplementation. There are a few studies showing no effect of dietary phytase supplementation on selected minerals [44, 45]. Furthermore, although serum Ca concentration decreased after phytase supplementation when compared to the Ca25% group and bone Ca and P content increased in both Cu-supplemented groups, the plasma and the bone Ca/P ratios remained unchanged. Probably, the dietary P and Ca concentration, bird breed, and age impacted Ca retention after phytase supplementation while having no effect in P retention. Similar observation was made by Chung et al. [43].

Our broiler chickens from the Cu25% + phyt group had longer and heavier tibiae with an increased bone weight/bone length ratio. Different results were obtained in the other study with the Cu-deficient chickens. Here, no difference was observed in the body weight or in the basal morphology of tibia between chickens fed the Cu-poor diet and of those from the Cu-deficient control group [46]. There is also another study conducted with Cu-Gly given to growing chickens throughout a 6-week period which showed that the reduction of dietary copper to 25% of the recommended level resulted in an increase in bone’s weight but without changes to its length [15].

In our study, a significant alteration was noted in bone geometry, which was dependent on phytase supplementation. Our broilers from the Cu25% + phyt group had more mature bones with a greater cross-sectional area of the midshaft which resulted in the increase of its mechanical strength and in decreased stresses. There was a 129 and 121% increase in the ultimate strength of the bones in birds supplemented with phytase (Cu25% + phyt), compared to the control Cu-deficient (0 Suppl) or Cu-poor diet without the addition of phytase (Cu25%), respectively. Significantly increased value in yield strength revealed an increase to the extent of elastic deformation after phytase supplementation. However, although bones of birds which form the Cu25% + phyt group were deforming and breaking under significantly higher loads, the reduced values of yield and ultimate stresses showed that tibiae from both Cu-supplemented groups were subjected to lower mechanical stresses before fractures as compared to the control 0 Suppl group. In conclusion, it can be assumed that the supplementation of Cu-Gly, even at lowered levels, positively influenced bone metabolism resulting in more mature bone and better mechanical strength in growing broilers. It is in agreement with results from another study performed on Cu-deficient chickens [4]. The bones of birds from the Cu-deficient control group had lower tolerance to deformation and were able to absorb less energy before fracture. Moreover, they demonstrated a reduced range of plastic deformation. However, even the lowest level of supplementation (2 ppm) was shown to be sufficient in preventing the bone changes seen in the Cu-deficient birds [4]. It seems that the amount of Cu needed to maintain the mechanical integrity of bone is substantially less than that required for growth [4]. Another study showed that after supplementing the diet with phytase, the tibia became stronger [41]. It is suggested that phytase supplementation results in better mineralization by improving dietary quality through the release of other trace minerals [17]. However, in contrast to our study, diets described contained higher level of phytase (600–1050 FTU kg^−1^) and different level of phosphorus [17, 41].

More detailed comparison of our findings with those from other studies is somewhat complicated, as no other studies specifically into the effects of dietary phytase supplementation in Cu-poor diets are available. Our study has shown improvement of the bone development in all Cu-Gly-treated broilers. However, the reserve (I), proliferative (II), and hypertrophic (III) zones in the tibia’s growth plate cartilage of the Cu25% + phyt chickens were wider when compared to other birds. The histological variation in birds with Cu deficiency suggested a defective or disorganized proliferation and mineralization within the analyzed zones of the growth plate. Thus, decreased bone mechanical resistance observed in the control 0 Suppl group could be linked with a failure in the mineral deposition within the extracellular matrix of the bone. Furthermore, the chickens fed the control 0 Suppl diet showed strongly osteoporotic cancellous bone. The widened reserve and hypertrophy zones observed in the Cu25% + phyt group could be due to the increased number of differentiating chondrocytes that resulted in the accumulation of cells within particular zones after Cu and phytase supplementation. Also, an increase in bone ash mineral content and the trabecular number relating to the supplementation of phytase and Cu-Gly were observed in the present study. Thus, it is reasonable to speculate that the association of osteocalcin with an increased cell proliferation and maturation in the growth plate of our phytase-treated chickens mediated by the increase of IGF-1 concentration could be the main cause of the improvement of bone development. The results obtained in the current study also indicate that the positive effects of phytase inclusion in the Cu-Gly diet on growth and development were mediated by improved activity of the somatotropic axis, as bone and cartilage homeostasis is regulated by GH acting via IGF-1 stimulator of the proliferation which was increased by 37% in the Cu25% + phyt group. Some studies suggest an important role of nutrition in the regulation of serum hormones like growth hormone, or IGF-1, one of somatomedins [47]. Nevertheless, the mechanism for exogenous enzymes—like phytase—on hormone regulation is problematic and requires further examination. Currently, there are a lack of studies relating to phytase supplementation and the somatotropic axis. However, the study on dietary supplementation with the enzyme xylanase reported an increase in the concentration of blood IGF-1 in 21-day-old broilers. This indicates that enhanced digestion and absorption of nutrients caused by enzyme supplementation has effects on blood hormone concentration [48].

The non-collagenous proteins contribute to a variety of functions within the bone such as matrix stabilization, calcification, and other metabolic regulatory activities. Approximately 10% of different non-collagenous proteins are expressed by osteoblasts and chondrocytes, and expression of these proteins is considered to be a specific marker [49, 50]. Neither the proteoglycan content nor the alteration in collagenous fibers in the articular cartilage was reported in broiler chickens after nutritional modification with phytase supplementation. Based on the obtained results, it can be concluded that both the presence of Cu in the diet and the phytase inclusion significantly enhanced the content of proteoglycans in the articular cartilage (Fig. 4). The phytase addition could exert a protective effect on chickens’ articular cartilage in which degenerative changes will occur much later when animals are fed the Cu-deprived or Cu-poor diet, as was shown in the morphometry and the distribution of proteoglycans (Fig. 4). The phytase inclusion improved the morphology of the articular cartilage, and its beneficial effect was particularly manifest in the widening of the superficial (I) zone. This modification can positively alter the load distribution in the joint and improve the elasticity of the articular cartilage, protecting it against the degradation in well-muscled and fast-growing broilers. Furthermore, the increase of the thickness of the transitional (II) zone can be equally beneficial, as it can also improve the transfer and distribution of the load through the joint. It can have other functional consequences, as the physical properties of articular cartilage are determined by the diversity in the components of the matrix and the difference in the quantitative mutual relations of collagen fibers. The content of proteoglycans plays an essential role in the destabilization of the collagen network [49]. The proportion of thin and thick fibers should give additional structural information about the influence of phytase addition on collagen synthesis in the articular cartilage (Fig. 5). The fine (green) collagen fibers might indicate an ongoing process of collagen synthesis occurring after Cu supplementation, the intensity of which was phytase-dependent (Fig. 5). Collagen is a major component of the extracellular matrix of many tissues, and its metabolism is directly associated with many physiological processes of biological adaptation. Moreover, the bone organic matrix contributes to bone elasticity and changes in the composition and spatial arrangement of soft bone tissues may also be responsible for the improved mechanical strength of bones observed in the experimented Cu-supplemented groups [25].

The present study also revealed many side effects of the Cu-deficient diet, e.g., very thin articular cartilage, especially the shortening of the superficial (I) zone. It can result in accelerated degradation of the articular cartilage linked with the loss of its elasticity causing difficulties in movement. Furthermore, it can provoke irreversible deformation caused by the impact of the load during movement. Finally, the diet devoid of Cu supplementation resulted in very low proteoglycan content in the articular cartilage (Fig. 4). Proteoglycans also provide stability to the articular cartilage, and the degradation of proteoglycans entails destabilization of the collagen network.

## Conclusions

To the best our knowledge, this is the first study that has examined both the mechanical properties of bone as well as the histomorphometry of cancellous bone and hyaline cartilage in chickens fed a diet low or deficient in copper but also a diet low in copper in relation to phytase supplementation. Irrespective of the phytase presence, the organic form of copper can lead to changes within the articular cartilage, as was indicated by the morphological analysis and the proteoglycan content. Phytase supplementation showed beneficial effects on differentiation in chicken growth plate chondrocytes, thereby sustaining their proliferative state and maintaining their sensitivity to growth factors, such as IGF-1. Furthermore, the phytase addition influenced the trabecular architecture of the tibia with subsequent improvement of its growth and development. However, the response of the skeletal system to phytase supplementation could differ among avian species due to differences in physiology of the digestive tract and gut microflora.

## References

[1] Hunt CD (1998). Copper and boron as examples of dietary trace elements important in bone development and disease. Curr Opin Orthop.

[2] Tomaszewska E, Dobrowolski P, Kwiecień M, Winiarska-Mieczan A, Tomczyk A, Muszyński S (2017). The influence of the dietary cu-glycine complex on the histomorphology of cancellous bone, articular cartilage, and growth plate as well as bone mechanical and geometric parameters is dose dependent. Biol Trace Elem Res.

[3] Rodríguez JP, Ríos S, González M (2002). Modulation of the proliferation and differentiation of human mesenchymal stem cells by copper. J Cell Biochem.

[4] Opsahl W, Zeronian H, Ellison M, Lewis D, Rucker RB, Riggins RS (1982). Role of copper in collagen cross-linking and its influence on selected mechanical properties of chick bone and tendon. J Nutr.

[5] Hong HH, Pischon N, Santana RB, Palamakumbura AH, Chase HB, Gantz D, Guo Y, Uzel MI, Ma D, Trackman PC (2004). A role for lysyl oxidase regulation in the control of normal collagen deposition in differetnting osteoblast cultures. J Cell Physiol.

[6] Tomaszewska E, Dobrowolski P, Kwiecień M (2016) Alterations in intestinal and liver histomorphology, and basal hematological and biochemical parameters in relation to different sources of dietary copper in adult rats. Ann Anim Sci. doi:10.1515/aoas-2016-0056 10.1007/s12011-015-0522-1 PMC4831993 26432448

[7] Yenice E, Mızrak C, Gültekin M, Atik Z, Tunca M (2015). Effects of organic and inorganic forms of manganese, zinc, copper, and chromium on bioavailability of these minerals and calcium in late-phase laying hens. Biol Trace Elem Res.

[8] Scott A, Vadalasetty KP, Sawosz E, Łukasiewicz M, Vadalasetty RKP, Jaworski S, Chwalibog A (2016). Effect of copper nanoparticles and copper sulphate on metabolic rate and development of broiler embryos. Anim Feed Sci Technol.

[9] Banks KM, Thompson KL, Rush JK, Applegate TJ (2004). Effects of copper source on phosphorus retention in broiler chicks and laying hens. Poult Sci.

[10] National Research Council (1994). Nutrient requirements of poultry.

[11] Bao YM, Choct M, Iji PA, Bruerton K (2007). Effect of organically complexed copper, iron, manganese and zinc on broiler performance, mineral excretion and accumulation in tissues. J Appl Poult Res.

[12] Tomaszewska E, Dobrowolski P, Kwiecień M, Burmańczuk N, Badzian B, Szymańczyk S, Kurlak P (2014). Alterations of liver histomorphology in relation to copper supplementation in inorganic and organic form in growing rats. Bull Vet Inst Pulawy.

[13] Tomaszewska E, Dobrowolski P, Kwiecień M (2016). Intestinal alterations, basal hematology and biochemical parameters in adolescent rats fed different sources of dietary copper. Biol Trace Elem Res.

[14] Świątkiewicz S, Koreleski J, Zhong DQ (2001). The bioavailability of zinc from inorganic and organic sources in broiler chickens as affected by addition of phytase. J Anim Feed Sci.

[15] Kwiecień M, Winiarska-Mieczan A, Zawiślak K, Sroka S (2014). Effect of copper glycinate chelate on biomechanical, morphometric and chemical properties of chicken femur. Ann Anim Sci.

[16] Sebastian S, Touchburn SP, Chavez ER (1998). Implications of phytic acid and supplemental microbial phytase in poultry nutrition: a review. World Poult Sci J.

[17] Aoyagi S, Baker DH (1995). Effect of microbial phytase and 1,25-dihydroxycholecalciferol on dietary copper utilization in chicks. Poult Sci.

[18] European Commission (2003). European Communities Directive no. 1334/2003 of the European Parliament and the Council. OJ.

[19] Aviagen (2014). Ross 308 broiler nutrition specification.

[20] AOAC (2000). Official methods of analysis of AOAC INTERNATIONAL.

[21] Frühbeck G, Alonso R, Marzo F, Santidrián S (1995). A modified method for the indirect quantitative analysis of phytate in foodstuffs. Anal Biochem.

[22] Žilić SM, Božović IN, Savić S, Šobajić S (2006). Heat processing of soybean kernel and its effect on lysine availability and protein solubility. Cent Eur J Biol.

[23] Tomaszewska E, Kwiecień M, Dobrowolski P, Klebaniuk R, Muszyński S, Olcha M, Blicharski T, Grela ER (2016) Dose-dependent effects of probiotic supplementation on bone characteristic and mineralization in female turkeys. Anim Prod Sci. doi:10.1071/AN16289

[24] Ferretti JL, Capozza RF, Mondelo N, Montuori E, Zanchetta JR (1993). Determination of femur structural properties by geometric and material variables as a function of body weight in rats. Evidence of sexual dimorphism. Bone.

[25] Muszyński S, Kwiecień M, Tomaszewska E, Świetlicka I, Dobrowolski P, Kasperek K, Jeżewska-Witkowska G (2017). Effect of caponization on performance and quality characteristics of long bones in Polbar chickens. Poult Sci.

[26] Tomaszewska E, Kwiecień M, Muszyński S, Dobrowolski P, Kasperek K, Blicharski T, Jeżewska-Witkowska G, Grela ER (2017) Long bone characteristics are altered by caponization and breed different. Br Poult Sci. doi:10.1080/00071668.2017.1280770 10.1080/00071668.2017.1280770 28102084

[27] Dobrowolski P, Tomaszewska E, Kurlak P, Pierzynowski SG (2016). Dietary 2-oxoglutarate mitigates gastrectomy–evoked structural changes in cartilage of female rats. Exp Biol Med.

[28] Tomaszewska E, Dobrowolski P, Kwiecień M, Wawrzyniak A, Burmańczuk N (2016). Comparison of the effect of a standard inclusion level of inorganic zinc to organic form at lowered level on bone development in growing male Ross broiler chickens. Ann Anim Sci.

[29] Bobinac D, Spanjol J, Zoricic S, Maric I (2003). Changes in articular cartilage and subchondral bone histomorphometry in osteoarthritic knee joints in humans. Bone.

[30] Tomaszewska E, Dobrowolski P, Winiarska-Mieczan A, Kwiecień M, Tomczyk A, Muszyński S (2017). The effect of tannic acid on the bone tissue of adult male Wistar rats exposed to cadmium and lead. Exp Toxicol Pathol.

[31] Tomaszewska E, Dobrowolski P, Siwicki AK (2012). Maternal treatment with dexamethasone at minimal therapeutic doses inhibits neonatal bone development in a gender-dependent manner. Livest Sci.

[32] Suvara SK, Layton C, Bancroft JD (2013). Bancroft’s theory and practice of histological techniques.

[33] Rich L, Whittaker P (2005). Collagen and picrosirius red staining: a polarized light assessment of fibrillar hue and spatial distribution. Braz J Morphol Sci.

[34] Mendes AS, Paixão SJ, Sikorski RR, Bonamigo DV, Morello MG, Ponzoni RAR (2016). Photogrammetry: a non-invasive and objective method for detecting locomotion problems in broiler chickens. Braz J Poult Sci.

[35] van Krimpen MM, de Jong IC (2014). Impact of nutrition on welfare aspects of broiler breeder flocks. World Poult Sci J.

[36] De Jong IC, Jones B, Bels V (2006). Feed restriction and welfare in domestic birds. Feeding in domestic vertebrates.

[37] Hocking PM, Jones EKM (2006). On-farm assessment of environmental enrichment for broiler breeders. Br Poult Sci.

[38] Kirkwood JK, Spratt DMJ, Duignan PJ, Kember NF (1989). Patterns of cell-proliferation and growth-rate in limb bones of the domestic-fowl (*Gallus-domesticus*). Res Vet Sci.

[39] Mench J, Weeks C, Butterworth A (2004). Lameness. Measuring and auditing broiler welfare.

[40] Tomaszewska E, Muszyński S, Dobrowolski P, Kwiecień M, Winiarska-Mieczan A, Świetlicka I, Wawrzyniak A (2017). Effect of zinc level and source (zinc oxide vs zinc glycine) on bone mechanical and geometric parameters, and histomorphology in male Ross 308 broiler chicken. Braz J Poult Sci.

[41] Qian H, Veit HP, Kornegay ET, Ravindran V, Denbow DM (1996). Effects of supplemental phytase and phosphorus on histological and other tibial bone characteristics and performances of broilers fed semi-purified diets. Poult Sci.

[42] Bao YM, Choct M, Iji PA, Bruerton K (2010). Trace mineral interactions in broiler chicken diets. Br Poult Sci.

[43] Chung TK, Rutherfurd SM, Thomas DV, Moughan PJ (2013). Effect of two microbial phytases on mineral availability and retention and bone mineral density in low-phosphorus diets for broilers. Br Poult Sci.

[44] Rutherfurd SM, Chung TK, Thomas DV, Zou ML, Moughan PJ (2012). Effect of a novel phytase on growth performance, apparent metabolizable energy, and the availability of minerals and amino acids in a low-phosphorus corn-soybean meal diet for broilers. Poult Sci.

[45] Um JS, Lim HS, Ahn SH, PaikK IK (2000). Effects of microbial phytase supplementation to low-phosphorus diets on the performance and utilization of nutrients in broiler chickens. Asian Australas J Anim Sci.

[46] Riggins RS, Cartwright AG, Rucker RB (1979). Viscoelastic properties of copper deficient chick bone. J Biomech.

[47] Hajati H, Rezaei M, Sayyahzadeh H (2009). The effects of enzyme supplementation on performance, carcass characteristics and some blood parameters of broilers fed on corn-soybean meal-wheat diets. Int J Poult Sci.

[48] Gao F, Jiang Y, Zhou GH, Han ZK (2008). The effects of xylanase supplementation on performance, characteristics of the gastrointestinal tract, blood parameters and gut microflora in broilers fed on wheat-based diets. Anim Feed Sci Technol.

[49] Tomaszewska E, Dobrowolski P, Bieńko M, Prost Ł, Szymańczyk S, Zdybel A (2015). Effects of 2-oxoglutaric acid on bone morphometry, densitometry, mechanics, and immunohistochemistry in 9-month-old boars with prenatal dexamethasone-induced osteopenia. Connect Tissue Res.

[50] Tomaszewska E, Dobrowolski P, Prost Ł, Hułas-Stasiak M, Muszyński S, Blicharski T (2016). The effect of supplementation of glutamine precursor on the growth plate, articular cartilage and cancellous bone in fundectomy-induced osteopenic bone. J Vet Med Sci.

